# Clinical Study of Vitamin D Levels in Hospitalized Children with Acute Respiratory Infections

**DOI:** 10.3390/pediatric16040088

**Published:** 2024-11-22

**Authors:** Gena Stoykova Petkova, Eleonora Nikolaeva Mineva, Venetsia Tsvetkova Botsova

**Affiliations:** 1 Department of Pediatrics, Medical University Pleven, 5800 Pleven, Bulgaria; 2 Dr. Georgi Stranski University Hospital, 5800 Pleven, Bulgaria; 3 Department of Social Medicine and Health Management, Faculty of Public Health, Medical University Pleven, 5800 Pleven, Bulgaria; eleonora.mineva-dimitrova@mu-pleven.bg

**Keywords:** vitamin D, respiratory system diseases, children

## Abstract

The aim of our research was to evaluate and analyze serum 25(OH) vitamin D and parathyroid hormone (PTH) levels to investigate whether vitamin D deficiency serves as a risk factor for an increased incidence of acute respiratory infections (ARI) in children. Serum PTH levels were used as an indicator of vitamin D sufficiency, as normal PTH levels require an optimal concentration of 25(OH) vitamin D. The study included 129 children, divided into five subgroups: children with acute bronchopneumonia (n = 42), acute laryngotracheitis (n = 7), acute bronchiolitis (n = 32), acute bronchitis (n = 18), and a control group (n = 30). No statistically significant differences in 25(OH)D levels were observed between the overall population of children with ARI and the control group (*p* = 0.073). However, significant differences in 25(OH)D levels were identified between the control group and children with bronchopneumonia, acute bronchitis, and laryngotracheitis (*p* < 0.01, *p* < 0.05). Regarding PTH levels, statistical significance was found between the control group and the acute bronchiolitis group, due to the high percentage of children with hypervitaminosis in this subgroup. These results highlight the crucial role of vitamin D in the onset and progression of acute respiratory tract infections in children, emphasizing its impact on their overall respiratory health.

## 1. Introduction

Acute respiratory infections (ARIs) are a major cause of morbidity in children worldwide, frequently leading to hospitalizations, particularly due to conditions such as bronchiolitis and bronchopneumonia [1,2,3]. Recently, there has been considerable debate regarding the role of vitamin D in immune responses to these infections and the potential need for supplementation. The majority of studies indicate that low vitamin D levels are widespread among infants and children with respiratory tract infections (RTIs). However, maintaining normal to elevated serum 25(OH)D (25-hydroxyvitamin D) levels appears to positively influence the incidence and severity of some, though not all, types of respiratory infections [4,5].

Vitamin D is a fat-soluble steroid primarily known for its essential role in regulating calcium-phosphorus metabolism, crucial for normal bone growth and development. However, its non-classical actions, mediated through vitamin D receptors (VDRs) expressed in various extrarenal tissues, are now well-documented. A key example is the immune system, where cells such as lymphoid, myeloid, dendritic cells, and macrophages increase VDR levels during acute infections. This promotes the production of active vitamin D, which, in turn, stimulates the generation of host defense proteins like cathelicidin and defensin. Additionally, vitamin D plays a role in innate immune signaling by promoting cytokine production and activating pattern recognition receptors (PRRs), which detect components of pathogenic microorganisms and trigger a rapid innate immune response [6,7]. These functions of vitamin D are crucial for local immunity in the lungs, as they are present not only in macrophages but also in lung epithelial cells [8]. This evidence underscores the role of vitamin D in the pathogenesis of certain lung diseases [9].

Vitamin D deficiency and insufficiency pose significant public health challenges globally, not only due to their involvement in various diseases but also because of the limited data on population-wide vitamin D status in many countries [10,11]. In Bulgarian children, few studies have investigated vitamin D status, and most focus on specific conditions such as idiopathic scoliosis, myopia, kidney and bowel disorders [12,13,14]. To date, few studies have explored its relationship with chronic respiratory infections and acute pneumonia [15,16]. Through our current research, we aimed to expand the data on vitamin D status in relation to respiratory infections in the Bulgarian pediatric population, focusing on various acute respiratory diseases. To achieve this, we included children with not only acute pneumonia but also bronchiolitis, laryngotracheitis, and acute bronchitis. Additionally, we aimed to compare our findings with existing data from studies conducted in different countries and populations, which are further analyzed in this article.

Calcidiol (25-hydroxycholecalciferol) is the primary vitamin D metabolite present in circulation, with a lifespan of 2–3 weeks, making its concentration the gold standard for assessing vitamin D status [17,18,19]. Measuring blood concentrations of 25-hydroxycholecalciferol and parathormone (PTH) allows for a precise evaluation of vitamin D status in children with acute respiratory infections. PTH levels serve as an indicator of vitamin D sufficiency, as normal PTH values require optimal levels of 25(OH) vitamin D [20].

The data obtained from these measurements aim to clarify the correlation between these laboratory parameters and the clinical manifestations of acute respiratory diseases in childhood. This study specifically sought to determine if there is a relationship between vitamin D levels and the frequency of certain acute infections of the respiratory tract in children.

## 2. Materials and Methods

This prospective clinical research was carried out at the Pediatrics Department of Dr. Georgi Stranski University Hospital, Pleven. A total of 129 children, aged 0–17 years, who were hospitalized due to acute respiratory infections (ARIs) between July 2021 and December 2023, were included in the study. The participants were divided into five subgroups:

- acute bronchopneumonia (n = 42),
- acute laryngotracheitis (n = 7),
- acute bronchiolitis (n = 32),
- acute bronchitis (n = 18), and
- control group (n = 30).

The children with acute bronchiolitis were between 2 months and 2 years of age.

Control subjects were healthy children who attended follow-up examinations at the clinic.

Exclusion criteria included: (1) children with acute non-infectious respiratory conditions (e.g., foreign body aspiration, congenital malformations); (2) those with chronic respiratory or circulatory system conditions (e.g., asthma, cystic fibrosis, primary ciliary dyskinesia, immunodeficiencies, congenital cardiovascular malformations); and (3) children with renal or endocrine diseases.

Serum 25(OH)D and parathyroid hormone (PTH) levels were measured using an electrochemiluminescent immunoassay on a Roche Cobas e411 immunological analyzer.

Vitamin D status was categorized based on the guidelines of the Bulgarian Society of Endocrinology as follows: vitamin D deficiency: <25 nmol/L, vitamin D insufficiency: 25–50 nmol/L, vitamin D sufficiency: 50–120 nmol/L, and hypervitaminosis D: >120 nmol/L. Serum PTH levels were classified as normal within the range of 15 to 65 pg/mL [21].

Statistical analysis was conducted using IBM SPSS Statistics v.26.0 for Windows, along with Microsoft Office Excel 2019. To determine where statistically significant differences in 25(OH)D levels existed between patients with specific diagnoses and the control group, the non-parametric Mann–Whitney U test was applied. A statistically significant difference was defined as a *p*-value < 0.05.

The study was approved by the Committee on Research Ethics at the Medical University—Pleven (Approval Code: 658; Approval Date: 29 June 2021). Informed consent for participation was obtained from the parents or guardians of all enrolled patients.

## 3. Results

A total of 129 children were enrolled in the study, consisting of 52 boys (40.3%) and 77 girls (59.7%). The mean age of the participants, calculated in months, was 31 ± 46.26, with an age range of 0 to 14 years.

**Figure 1:**
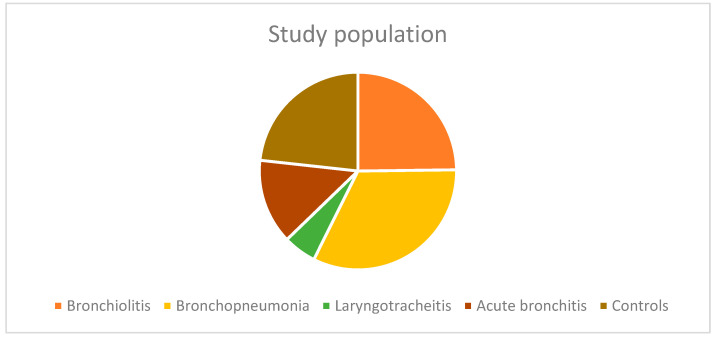
Distribution of the study population by group: acute bronchiolitis (n = 32), acute bronchopneumonia (n = 42), acute laryngotracheitis (n = 7), acute bronchitis (n = 18), and control group (n = 30).

Figure 1 illustrates the group distribution of the study population.

In our study, both subnormal levels of vitamin D and cases of hypervitaminosis were observed in the individual groups:

- In the acute bronchitis group, seven children (38.9%) were found to be insufficient in vitamin D, two (11.1%) were deficient, and one child (5.6%) had hypervitaminosis.
- In the group of children with laryngotracheitis, two (28.6%) exhibited vitamin D insufficiency, and one (14.3%) had vitamin D deficiency.
- Among children with bronchopneumonia, sixteen (38.1%) had vitamin D insufficiency, and four (9.5%) presented with hypervitaminosis. Vitamin D levels in the other twenty-two children in this group were found to be within the reference range.
- Abnormalities in vitamin D status were observed in children with acute bronchiolitis, with three (9.4%) showing insufficiency, one (3.1%) showing deficiency, and fourteen (43.8%) having hypervitaminosis.

**Figure 2:**
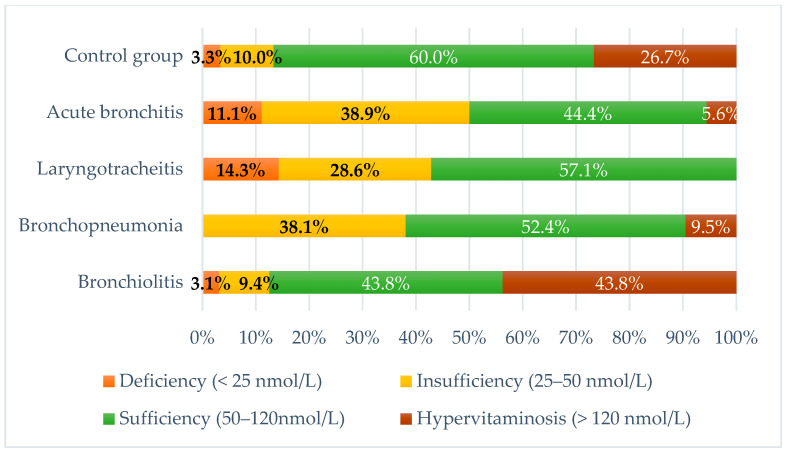
Distribution of the study population based on serum 25 (OH) D levels. *p* = 0.01.

The results are illustrated in Figure 2.

**Figure 3:**
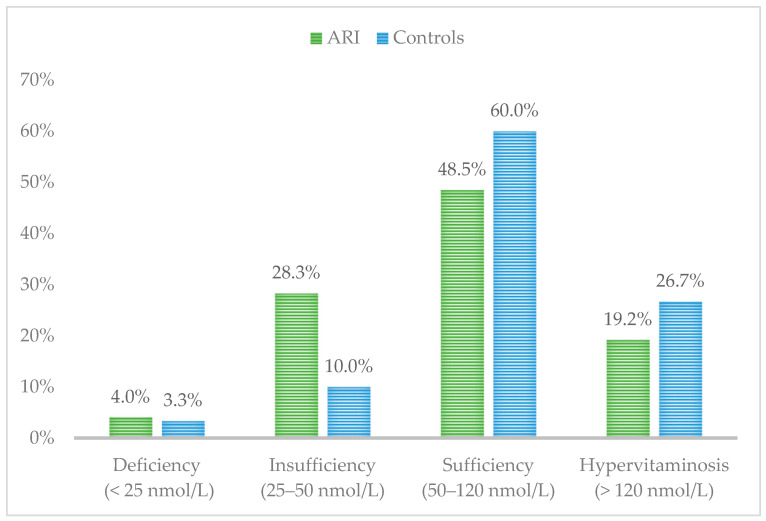
Vitamin D status in children with acute respiratory infections (ARIs) versus healthy controls.

Comparison of serum 25 (OH) D levels between children with ARIs and healthy controls revealed the following: 28.3% of ARI patients exhibited vitamin D insufficiency, compared to 10% of the healthy group. 4% of children with ARIs were found to have vitamin D deficiency, compared to 3.3% in the control group. Interestingly, a higher percentage of hypervitaminosis (26.7%) was observed among the healthy children (Figure 3). 

No statistically significant difference in 25-hydroxyvitamin D levels was observed between the general population of children with ARI (mean: 81.06 ± 47.018) and the control group (*p* = 0.073, U = 1163.000). However, a statistically significant difference in 25(OH)D levels was found between the control group and children with bronchopneumonia, acute bronchitis, and laryngotracheitis (Table 1).

Regarding PTH levels, a statistically significant difference was observed between the control group and the children with acute bronchiolitis, likely due to the high percentage of hypervitaminosis in this group (Table 2).

## 4. Discussion

Vitamin D deficiency and its consequences have long been a focus of scientific research. In recent years, the immunomodulatory effects of active vitamin D, driven by its local synthesis in the lungs, immune system cells, and other extrarenal tissues, have become increasingly understood [22]. There is evidence of widespread expression of the enzyme 1α-hydroxylase throughout the body, which catalyzes the final step in the synthesis of the active form of 1,25-dihydroxyvitamin D3 (1,25 D), along with the expression of vitamin D receptors (VDRs) that mediate its localized actions [23].

Regarding the association between low vitamin D serum levels and pediatric pulmonary pathology, systematic reviews up to 2019 reported that studies were limited and inconclusive [24]. However, in recent years, research on this topic has increased significantly. A recent meta-analysis from 2022 concluded that children with low vitamin D levels are more susceptible to developing respiratory infections [25]. Our study aims to contribute to the existing data on the relationship between specific acute respiratory infections in childhood and low vitamin D levels.

A 2017 systematic review examining vitamin D status in southern European countries found that, despite the higher availability of UVB radiation compared to northern Europe, vitamin D deficiency was still prevalent. The review also indicated that neonates, infants, and adolescents tend to have higher rates of vitamin D deficiency or insufficiency compared to adults and the elderly [26]. However, data on vitamin D status in Bulgarian children, particularly its connection to the incidence of acute respiratory infections, remain scarce.

One key challenge in our study was the lack of universal consensus and limited guidelines for defining vitamin D status in both children and adults [27,28,29]. The Institute of Medicine and the European Calcified Tissue Society define vitamin D deficiency as serum 25-hydroxyvitamin D (25(OH)D) levels below 50 nmol/L, severe deficiency as levels below 30 nmol/L, and vitamin D insufficiency as levels between 52.5 and 72.5 nmol/L [30,31]. In our study, we followed the criteria recommended by the Bulgarian Society of Endocrinology for adults, which define vitamin D deficiency as <25 nmol/L and insufficiency as 25–50 nmol/L. However, many of the studies we referenced used criteria from the aforementioned organizations or different ones, which may lead to potential confusion and varying interpretations of the data.

In our study, 28.3% of children with acute respiratory infections and 10% of the control group had vitamin D levels below 50 nmol/L, classified as insufficiency by the Bulgarian Society of Endocrinology. Although the difference in deficiency (<25 nmol/L) percentages was not as large, it was still more prevalent in the case group (4%) compared to the control group (3.3%). These results are similar to those in a study of Nigerian children with ARIs, which reported serum vitamin D levels < 50 nmol/L in 10.8% of cases compared to 3.2% in controls [32]. Comparable findings were reported in a study conducted in China, which investigated hospitalized children with various acute respiratory infections, as well as another study on children with community-acquired pneumonia, both finding vitamin D levels below 50 nmol/L. However, in these studies, the results were interpreted as vitamin D deficiency, while in our study, they were classified as insufficiency [33,34]. An Indian study reported that three-fourths of children with recurrent acute respiratory infections were vitamin D deficient, using a cutoff value of <50 nmol/L for deficiency [35].

Surprisingly, 26.7% of healthy children had vitamin D levels above 120 nmol/L, which we classified as hypervitaminosis in this study. Fortunately, no clinical signs of intoxication were observed in these patients. Moreover, according to the aforementioned studies, these levels could be interpreted as sufficient, as vitamin D toxicity typically occurs when serum 25(OH)D levels exceed 375 nmol/L (150 ng/mL) [36].

We observed no statistically significant difference in 25(OH)D levels between the general population of children with ARIs and the control group, primarily due to the considerable proportion (43.8%) of children with hypervitaminosis in the acute bronchiolitis group. Additionally, only 9.4% were found to be insufficient, and 3.1% presented with vitamin D deficiency. These findings differ from recent studies, which reported vitamin D insufficiency rates of 47.8%, 62.5%, and 73% in different cohorts of children with acute bronchiolitis [37,38,39]. The acute bronchitis, laryngotracheitis, and pneumonia groups exhibited the highest percentages of vitamin D insufficiency (<50 nmol/L), with a statistically significant difference in 25(OH)D levels compared to the control group.

Lastly, this study has several limitations. The distribution of patients across the study groups was uneven, as enrollment was based on the respiratory conditions presented at the clinic during the study period. Furthermore, the number of patients was constrained by the available institutional funding, which limited the sample size. Due to these financial constraints, we were unable to monitor the markers over time and could only measure them once, during the initial phase of infection.

We believe that to better understand the role of vitamin D status in ARI during childhood, its relationship with inflammatory markers (CRP, pro-inflammatory cytokines, and WBC count) should be further explored, which we plan to address in a future study.

## 5. Conclusions

Our study found that low serum 25(OH)D levels are associated with specific acute respiratory diseases in children, including bronchopneumonia, acute bronchitis, and laryngotracheitis. Therefore, we believe that the immunomodulatory effects of vitamin D warrant close attention. Ongoing evaluation of serum 25(OH)D levels in children with acute respiratory infections is necessary to better understand the true correlation between vitamin D status and these infections. This knowledge will be essential for developing effective therapeutic strategies to manage and prevent acute respiratory infections in the future.

Maintaining sufficient vitamin D levels is crucial for the health of pediatric patients, and supplementation ought to be provided when there is insufficiency or deficiency.

## Figures and Tables

**Table 1:** Mean levels of 25-hydroxyvitamin D in the five studied child groups.

Group	N	25(OH)D nmol/L	
		Mean	SD
Bronchiolitis	32	114.4	51.8
Bronchopneumonia	42	67.0 **	36.3
Laryngotracheitis	7	64.5 *	33.2
Acute bronchitis	18	61.0 **	34.1
Control group	30	97.4	54.7
Total	129	84.8	49.2

** *p* < 0.01, * *p* < 0.05.

**Table 2:** Average values of parathyroid hormone (PTH) in the five groups of examined children.

Group	N	PTH pg/mL	
		Mean	SD
Bronchiolitis	32	8.5 *	7.1
Bronchopneumonia	42	24.8	18.4
Laryngotracheitis	7	19.5	8.0
Acute bronchitis	18	23.2	14.7
Control group	30	23.1	15.9
Total	129	19.9	15.9

* *p* < 0.001.

## Data Availability

This article does not involve data sharing, as there were no new data created or analyzed during the study.
